# Peptides mimicking viral proteins of porcine circovirus type 2 were profiled by the spectrum of mouse anti-PCV2 antibodies

**DOI:** 10.1186/s12865-017-0211-2

**Published:** 2017-05-15

**Authors:** Ling-Chu Hung, Cheng-Yao Yang, Ivan-Chen Cheng

**Affiliations:** 1 Animal Health Research Institute, Council of Agriculture, Executive Yuan, No.376, Zhongzheng Rd., Danshui Dist., New Taipei, 25158 Taiwan; 2 Livestock Research Institute, Council of Agriculture, Executive Yuan, No.112, Muchang, Xinhua Dist., Tainan, 71246 Taiwan; 3 School of Veterinary Medicine, National Taiwan University, No.1, Sec. 4, Roosevelt Road, Taipei, 10617 Taiwan; 4 Agricultural Technology Research Institute, No.52, Kedong 2nd Rd., Zhunan Township, Miaoli, 35053 Taiwan

**Keywords:** Porcine circovirus type 2, Peptide, Mimic, Open reading frame proteins, Antibody

## Abstract

**Background:**

Porcine circovirus 2 (PCV2) is a small, non-enveloped DNA virus causing swine lymphocyte depletion and severe impact on the swine industry. The aim of this study was to evaluate the antigenicity and immunogenicity of specific peptides, and seeking the potential candidate of PCV2 peptide-based vaccine. It’s initiating from peptides reacting with PCV2-infected pig sera and peptide-immunized mouse sera.

**Results:**

The data showed that the sera from PCV2-infected pigs could react with the N-terminal (C1), middle region (C2), and C-terminal peptide (C3) of PCV2 capsid protein (CP), ORF3 protein (N1), ORF6 protein (N2) and ORF9 protein (N3). This study demonstrated that anti-PCV2 mouse antisera could be generated by specific synthetic peptides (C3 and N2) and recognized PCV2 viral protein. We found that the tertiary or linear form C-terminal sequence (C3) of PCV2 capsid peptide only appeared a local distribution in the nucleus of PCV2-infected PK cells, virus-like particles of PCV2 major appeared a local distribution in the cytoplasm, and ORF 6 protein of PCV2 were shown unusually in cytoplasm. Furthermore, most residues of the C1 and the C3 were presented on the surface of PCV2 CP, in the view of 3-D structure of the CP. Our data demonstrated that PCV2-infected pigs had higher OD_405_ value of anti-C3 IgG on Day 1, Month 3 and Month 6 than in Month 1. These pigs had higher anti-C3 IgM level in Month 3 and Month 6 than on Day 1 (*P* < 0.01).

**Conclusions:**

We demonstrated that the key peptide (C3) mimic the C-terminal of PCV2 capsid protein which were capable of inducing antibodies. The specific antibody against the C3 were confirmed as the serological marker in PCV2-infected pigs.

**Electronic supplementary material:**

The online version of this article (doi:10.1186/s12865-017-0211-2) contains supplementary material, which is available to authorized users.

## Background

Porcine circovirus (PCV) is the smallest non-enveloped icosahedral viruses, and contains circular single-stranded DNA [1]. Porcine circovirus type 1 (PCV1) is considered to be non-pathogenic to pigs [2]; however, Porcine circovirus type 2 (PCV2) is recognized as one of the most important viral pathogens in modern swine production, and an endemic disease in pig herds [3, 4]. It is associated with a number of manifestations, including post-weaning multisystemic wasting syndrome (PMWS), porcine dermatitis and nephropathy syndrome (PDNS), respiratory disease, lymphadenopathy, enteritis, and reproductive disease [5–7].

The two types of PCVs, both contain 11 potential open reading frames (ORFs) [8]. ORF1 and ORF2 genes are the two major ORFs and orientated in opposite directions. ORF1 encodes for the non-structural replicase proteins Rep and Rep’ [8, 9]. The structural capsid protein (CP) is only encoded by ORF2 [8, 10]. The ORF3 non-structural protein of PCV2 interacts with Pirh2, an E3 ligase involved in the ubiquitination of p53, resulting in the decreased levels of Pirh2 and an increase in levels of p53, further leading to apoptosis of the virus-infected cells and enhance the spread of the virus [11–15]. The ORF4 gene is expressed at the level of transcription in the PCV2-infected cells [16], however, ORF4 protein is not essential for PCV2 replication but plays a role in suppressing caspase activity and regulating CD4+ and CD8+ T lymphocytes during PCV2 infection [17]. The ORF5 protein is not essential for PCV2 replication and likely plays an important role in regulating the NF-κB signaling pathway [18]. Furthermore, ORFs 5, 6, 9, 10, and 11 in these two circoviruses (PCV1 and PCV2) lack any homology with any other ORF. These predicted differences in proteins, encoded in PCV2, are possibly the contributing factors for the pathogenesis, clinical signs, and lesions associated with PMWS [8]. To date, proteins of ORFs 6, 9, 10, and 11 have not yet been determined whether these predicted proteins exist or whether they elicit any humoral immune response.

PCV2 can be divided into PCV2a and PCV2b genotypes which are present worldwide. A third genotype, PCV2c, has only been identified in Danish archived samples from pigs [19, 20]. According to the classification system of previous studies [19, 21, 22], PCV2 strains were also defined as two subgroups with eight clusters: PCV2b-1A to PCV2b-1C and PCV2a-2A to PCV2a-2E. Some capsid peptides could be used as immunorelevant epitopes for virus strains discrimination. Antigenic domains in the capsid protein of PCV2 (residues 65–87, 113–139 and 193–207) were demonstrated by PEPSCAN analysis [23]. Two peptides (residues 25–43 and 169–183) common to ORF2 from PCV1 and PCV2 could also be identified in this way, although variations could be observed depending on the serum used [23]. It has been previously reported that PCV1/PCV2 chimeric viruses and PCV2 monoclonal antibodies (mAbs) were utilized to determine antigenic epitopes of the capsid protein of PCV2 (PCV2 CP) [24]. Their data showed that at least five different but overlapping conformational epitopes within residues 47–63, 165–200 and 230–233 of the PCV2 CP. Linear B-cell epitopes within the residues 156–162, 175–192, 195–202, and 231–233 of the PCV2 CP were demonstrated by peptide-based ELISA approaches and mAbs [25]. The conformational epitope of CP was composed of the motif the residues 231–233 and 1–60 together [25]. The residues 26–36 were identified as an antigen epitope in the nuclear localization signal region of PCV2 CP [26]. Some studies demonstrated that the residues 59 and 60 of PCV2 CP participated in the formation of conformational neutralizing epitopes and mutations at residues 59 or 59/60 formed a novel neutralizing epitope for mAb 8E4-negative strains [27, 28]. The decisive residues 59, 63, 83, 130, 133, 206, 210 of CP are responsible for the interactive binding of mAbs with different PCV2 strains [22].

Recently, more and more researchers try to generate cutting-edge viral vaccines, most of designer selected critical epitopes of viral protein as potential candidates for the vaccine [29–31]. This study investigated the antigenicity and immunogenicity of couples of synthetic peptides, for seeking potential candidate PCV2 peptide-based vaccine. Based on previous studies, it is probably that antibodies prefer bind to those epitopes rather than non-epitopes only on PCV2 CP. If this hypothesis is true, it will be feasible to utilize synthetic peptide consisting of these epitopes to elicit antibodies which could recognize native proteins of PCV2 CP. Therefore, the synthetic peptides were analyzed for the binding with field sera collected from PCV2-infected herds. The data showed field sera can react with the peptides of PCV2 CP (ORF2 protein), ORF3 protein, ORF6 protein and ORF9 protein. Here, we explored those peptides could be new candidates of immunogen involved in humoral immunity. To demonstrate the peptides can mimic the epitopes present on the native PCV2 CP or non-structural proteins, we utilized the conjugated peptide-KLH to inoculate mice and test their sera. In another way, this study was also to investigate anti-peptides specific antibodies titers in the serum of piglets during 6 months of the postnatal period.

## Methods

### Peptides

Peptides were synthesized by the solid-phase peptide synthesis method using a ThuraMed Tetras106 Peptide Synthesizer (CreoSalus, USA). Peptide purity was assessed by high performance liquid chromatography (LC-10ATVP serial dual plunger pump, Shimadzu, USA) and were tested for the correct mass by Waters Micromass ZQ™ 2000 LC Mass Spectrometer (Waters, USA). All synthetic peptides used in this study were synthesized by the Yao-Hong Biotechnology Inc. (New Taipei, Taiwan) and listed in Table 1. Six synthetic peptides (as shown in Table 1) included the N-terminal sequence of PCV2b CP between residues 59 and 86 (C1), the middle region sequence of PCV2b CP between residues 108 and 136 (C2), the C-terminal sequence of PCV2b CP between residues 195 and 233 (C3), the N-terminal sequence of PCV2b ORF3 protein between residues 35 and 66 (N1), the full sequence of PCV2 ORF 6 protein between residues 1 and 29 (N2), and the full sequence of PCV2 ORF 9 protein between residues 1 and 42 (N3). Some peptides (C2 and N3) already had cysteine in their sequence, while others (C1, C3, N1, and N2) were appended with an N-terminal cysteine during synthesis, which was required for conjugation with maleimide-activated carriers. In order to induce specific antibodies against these peptides of PCV2 proteins, six peptides (C1, C2, C3, N1, N2, and N3) were respectively conjugated to keyhole limpet hemocyanin (KLH) using heterobifunctional cross-linker Sulfo-SMCC (Thermo scientific, Rockford, IL, USA).

**Table 1:** Design of synthesized peptides sequence of PCV2 capsid protein, and non-capsid proteins

Peptide name	PCVType	Position	Peptide sequence
C1	2b	CP59-86	CRTTVKTPSWAVDMMRFNINDFLPPGGGS
C2	2b	CP108-137	CSPITQGDRGVGSSAVILDDNFVTKATALT
C3	2b	CP195-233	CHVGLGTAFENSIYDQEYNIRVTMYVQFREFNLKDPPLNP
N1	2b	ORF3 (35–66)	CHNDVYISLPITLLHFPAHFQKFSQPAEISDKR
N2	2	ORF6 protein	CMASSTPASPAPSDILSSEPQSERPPGRWT
N3	2	ORF9 protein	MGLGSASSILLAGHVAAEVLPRCCRCRSALVILTAHEFRFQI

### Field sera collected from a PCV2-unvaccinated conventional farrow-to-finish herd

The study farm was located in Tainan, Taiwan. All pigs were notched in their ear with a unique identification number on the day of birth and weaned at 4 weeks of age and transferred into nursery houses. At about 8 weeks of age they were moved to the fattening house. Piglet were routinely vaccinated with commercial *Mycoplasma hyopneumoniae*, atrophic rhinitis & *Pasteurella multocida*, *Actinobacillus pleuropneumoniae*, erysipelas, hog cholera (HC), pseudorabies (g1 negative), and foot-and-mouth disease (FMD) vaccines according to each manufacturer’s recommendation. Sows were vaccinated against pseudorabies and colibacilosis. This conventional pig farm had been PCV2 infection. Pig sera were detected seropositive for PCV2 by indirect fluorescence antibody (IFA) assay with Porcine Circovirus FA Substrate Slide (VMRD, USA), and confirmed as positive for PCV2 DNA by PCR. However, this farm did not immunize pigs against PCV2.

From each litter, two newborn piglets (1 male & 1 female) were picked, whose body weights were close to the mean body weight of the entire newborn litter. The study involved 22 newborn piglets of TLRI Black Pig No.1 (TBP), delivered from 11 sows during 4 seasons of one year. Blood samples from each pig were collected 4 times during this experiment: on the 1st day (after colostrum uptake), 1st month, 3rd month, and 6th month of life, and those sera were stored at -20 °C. The care and use of pig was approved by the Institutional Animal Care and Use Committee, Livestock Research Institute (LRI), Council of Agricultural, Taiwan to ensure the compliance of the local legal and ethical requirements.

### PCV2-negative sera

PCV2-negative sera were collected from the PCV2-negative herd in the primary specific pathogen free (SPF) pig facility, Agricultural Technology Research Institute. All piglets were delivered by surgical process with Caesarian-section and send into sterile isolation cage and raised on sterile milk replacer. Serum samples were collected from 20 pigs (4 each from 1-week, 1-month, 2-month, 3-month, and 6-month old pigs). Those serum samples were confirmed negative for PCV2 nucleic acids by PCR detection and antibodies of PCV2 were also negative by IFA assay with Porcine Circovirus FA Substrate Slide.

### Immunoreactivities of peptides with antisera from a PCV2-unvaccinated conventional farrow-to-finish herd

Immunoreactivities of synthetic peptides and virus-like particles (VLP) of PCV2 (CircoFLEX®, Boehringer Ingelheim, USA) were determined using an iELISA with sera from a PCV2-unvaccinated conventional farrow-to-finish herd. Ninety-six-well Maxisorp plates were coated with 100 μL of one PCV2 peptide (5 μg/ml of peptide or 15 μL/ml of VLP of PCV2) in bicarbonate buffer and incubated overnight at 4 °C. After three washes with PBS containing 0.05% Tween 20 (PBST), the plates were blocked with 100 μL of PBST containing 5% casein hydrolysate for 30 min at 37 °C. After washing, 100 μL of 1:100 diluted pig sera with PBST containing 0.5% BSA (PBST-B) were added and plates were again incubated for 2 h at 37 °C. After rinsing three times with PBST, 100 μL of peroxidase-conjugated goat anti-swine IgG γ chain (KPL, Gaithersburg, MD, USA) in PBST-B was added, and then incubated at 37 °C for another 1 h. The plates were then washed three times, and the colorimetric reaction was developed using 100 μL of chromogenic substrate ABTS (Sigma-Aldrich, USA). Following a 30-min incubation away from light, absorbance values were read at 405 nm using a SpectraMax M5 microplate reader (Molecular Devices, USA).

### Preparation of mouse antisera against the PCV2 peptides

Five-week-old, female, BALB/cByJNarl (BALB/c) mice were purchased from a specific pathogen free (SPF) colony (the National Applied Research Laboratories, Taiwan). SPF status was verified by bacteriology, parasitology, histopathology, serology, and genetic testing through the supplier and no specific pathogens were detected. Mice were maintained in isolation rooms in filtertop cages and the room temperature was at 20–26 °C. Mice were fed with a commercially pelleted diet (rodent chow), and pure water was available ad libitum. This study follow the standards of the Guide of the Care and Use of Laboratory Animals and the study protocol was approved by the Committee of Animal Experimentation of Livestock Research Institute, Council of Agriculture, Taiwan, and the Committee of Animal Experimentation of Animal Health Research Institute, Council of Agriculture, Taiwan. All BALB/c mice exhibited low background anti‐peptide IgG by iELISA before immunization, which was acceptable for further immunization with the conjugated peptide (Table 1). Then four mice per group were immunized subcutaneously with a given conjugated peptide or VLP of PCV2 three times at 2-week intervals. For the primary immunization per mouse, 65 μg conjugated peptide was mixed with 0.15 mL complete Freund’s adjuvant (Sigma-Aldrich, USA). For secondary and third immunization, the same amount of immunogens as the initial injection were mixed with incomplete Freund’s adjuvant (Sigma-Aldrich, USA). The mice in the PCV2 vaccine group were injected intramuscularly in legs with 0.1 ml of the commercial vaccine (CircoFLEX®, Boehringer Ingelheim). Two weeks after last booster, 0.05–0.1 mL of blood was collected via the tail vein. The serum was separated, and anti‐peptide antibody titer in each sample was measured by iELISA.

### IFA

The Porcine Circovirus Type 2 (PCV-2, T657 strain) FA substrate slides (Cat No. SLD-IFA-PCV2, Lot. P140313-001, VMRD, USA) were incubated with a 1:100 dilution of antiserum from experimentally peptide-immunized mice, and a 1:100 dilution of PCV2 convalescent-phase swine antiserum. After incubation at 37 °C for 1 h, slides were gently rinsed briefly in PBS and then soak for 15 min in PBS at 4 °C. The slides were then incubated at 37 °C with fluorescein isothiocyanate (FITC)-labeled goat anti-mouse IgG (subclasses 1 + 2a + 2b + 3, Fcγ), FITC- labeled goat anti-pig IgG (H + L) (Jackson Immunoresearch, West Grove, PA, USA). 30 min after incubation, slide were washed with PBS and then incubated with 4, 6-diamidino-2-phenylindole (DAPI) (AAT Bioquest, Sunnyvale, CA, USA) at a dilution of 1 in 2300 in PBS for 15 min at room temperature. Samples were mounted under 50% glycerol and observed with an Olympus BX51 fluorescence microscope and SPOT FlEX camera (Diagnostic Instrument, Model 15.2 64MP, USA).

### Western blotting

Imumunospecificities of mouse antisera and mAbs were assayed against VLP of PCV2 (CircoFLEX®) by Western blotting. VLP of PCV2 is based on an open reading frame 2 (capsid) protein expressed in the baculovirus system and spontaneously forms a VLP. VLP of PCV2 were separated by Bolt^TM^Bis-Tris Plus Gel (Invitrogen, Carlsbad, CA, USA) on 10% polyacrylamide and then transferred to a polyvinylidene difluoride (PVDF) membrane (Millipore, USA) in Fast Semi-Dry transfer buffer (Thermo, USA) using a Yrdimes Semi-dry transfer system (Wealtec, Taiwan) at 0.5 mA/cm^2^ membrane for 10 min. The membrane was blocked with PBST containing 5% casein hydrolysate for 30 min at 37 °C, and then incubated with mouse anti-PCV2 sera, or mAbs to capsid protein of PCV2 at 37 °C for 2 h, respectively. After three washes in PBST, the membranes were incubated with the secondary Abs, horseradish peroxidase (HRP)-conjugated goat anti-mouse IgG (subclasses 1 + 2a + 2b + 3, Fc_γ_) (Jackson Immunoresearch, USA) at 37 °C for 1 h. Wash three times with PBST, 5 min each wash. The membranes were incubated with TOPBIO Enhanced chemiluminescence (ECL) substrate (Topbio, Taiwan) for 5 min. Images were captured in a MultiGel-21-C2 chemiluminescent imaging system (Topbio, Taiwan), and quantified with the ImageJ software (NIH, USA).

### Swine isotype-specific antibody immunoassay for C3

Ninety-six microtiter plates (Nunc, Rochester, NY, USA) were coated with 100 μl of the peptide C3 (5 μg/ml) in 0.05 M bicarbonate buffer and incubated overnight at 4 °C. After three washes with PBST, the plates were blocked with100 μL of PBST containing 5% casein hydrolysate for 30 min at 37 °C. After washing, a sequential 10-fold dilution of serum samples with PBST-B was added, each sample in duplicate, and plates were again incubated for 2 h at 37 °C. After rinsing three times with PBST, 100 μL of peroxidase-conjugated goat anti-swine IgG γ chain or IgM μ chain (KPL, Gaithersburg, MD, USA) in PBST-B was added in order to detect C3-specific IgG or IgM antibodies, and then incubated at 37 °C for another 1 h. The plates were then washed three times, and the colorimetric reaction was developed using 100 μL of ABTS. Following a 30 min incubation away from light, absorbance values were read at 405 nm using a SpectraMax M5 microplate reader.

We want to detect swine specific IgA antibody, however, we cannot find an available secondary antibody (HRP-conjugated anti-swine IgA). For this reason, we use the alternative method (sandwich way) to use mouse anti-pig IgA α chain mAb and then add HRP-conjugated goat anti-mouse IgG. Briefly, Ninety-six microtiter plates were coated with 100 μl of the peptide C3 (5 μg/ml) in 0.05 M bicarbonate buffer and incubated overnight at 4 °C. After three washes with PBST, the plates were blocked with100 μL of PBST containing 5% casein hydrolysate for 30 min at 37 °C. After washing, a sequential 10-fold dilution of serum samples with PBST-B was added, each sample in duplicate, and plates were again incubated for 2 h at 37 °C. After rinsing three times with PBST, 100 μL of mouse anti-pig IgA α chain mAb (AbD Serotect, UK) in PBST-B was added in order to detect C3-specific IgA antibodies, and then incubated at 37 °C for another 1 h. After washed three times and subsequently incubated for another 1 h at 37 °C with HRP-conjugated goat anti-mouse IgG (subclasses 1 + 2a + 2b + 3, Fcγ, minimal cross- reaction to other animals serum protein, Jackson ImmunoResearch, USA). The plates were then washed three times, and the colorimetric reaction was developed using 100 μL of ABTS. Following a 30 min incubation away from light, absorbance values were read at 405 nm using a SpectraMax M5 microplate reader.

### Sequence alignments

Six different PCV2 strains from different PCV2 genetic clusters have been previously described in Saha et al [22]. Amino acid sequence alignment for residues 1 to 233 (or 234) in the PCV2 CP was used in this study. Multiple alignments of these sequences were performed using the T-Coffee multiple-alignment tool [32] and displayed with Jalview Version 2 [33].

### Protein structural images

The three-dimensional structures of the capsid protein were used as approaches to figure out the interactive binding of peptides and antibody. The structural model of the capsid protein coordinates were retrieved from the Protein Data Bank (PDB) entries for the PCV2 CP (PDB code: 3R0R), and images were generated using UCSF Chimera version 1.6.2 [34] from the Resource for Biocomputing, Visualization, and Informatics at the University of California, San Francisco, USA.

## Results

### Immunoreactivities of peptides with swine sera

**Fig. 1:**
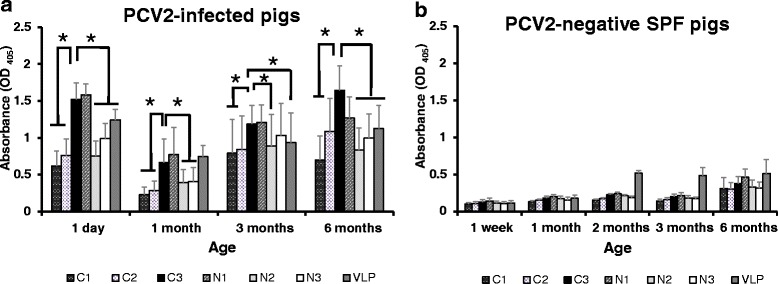
Immunoreactivities of synthetic peptides were carried out with those sera from unvaccinated naturally PCV2-infected pigs in comparison with sera from PCV2-negative pigs. **a** In the unvaccinated naturally PCV2-infected pig farm, 22 newborn piglets of TBP, were delivered from 11 sows during 4 seasons of 1 year. Blood samples from each pig were collected 4 times at aged 1 day (after colostrum uptake), 1 month, 3 months and 6 months. **b** 20 PCV2-negative sera (4 each from 1-week, 1-month, 2-month, 3-month, and 6-month old pigs) were collected from the PCV2-negative herd in the primary specific pathogen free (SPF) pig facility. Synthetic peptides were used in indirect ELISA to measure immunoreactivitiy with these pig sera (each serum dilution 1:100). All samples were assayed twice. Error bars indicate SD from the mean. Significant *p* values are indicated as *p < 0.05. Statistical significance was calculated using paired Student’s *t*-test

Immunoreactivities of synthetic peptides were carried out with field sera collected from the PCV2-infected herd in comparison with sera from the PCV2-negative herd. The results indicated that these field sera collected from the PCV2-infected herd had higher OD_405_ value of IgG (against peptides or VLP of PCV2) at aged 1 day, 3 months and 6 months, compared with aged 1 month (*p* < 0.05) (Fig. 1a). The detection levels (OD_405_ value of IgG against peptides or VLP of PCV2, excluding N1) in newborn (day 1) shown similar to 6 month olds in the PCV2-infected pigs. These field sera had significant higher OD_405_ value of IgG against C3 (or N1) than OD_405_ value of IgG against other peptides in every age (*p* < 0.05). These sera showed the lowest OD_405_ value of IgG against C1 compared with other peptides and VLP of PCV2 in every age. Further, these field sera had higher OD_405_ value of IgG (against peptides or VLP of PCV2) than that of PCV2-negative pig sera in every age (Fig. 1b). These PCV2-negative pig sera had higher OD_405_ value of IgG (against VLP of PCV2) than OD_405_ value of IgG against all peptides at aged 2 months and 3 months. These PCV2-negative pig sera showed higher OD_405_ value of IgG against all peptides at aged 6 months compared with aged 1 week, 1 month, 2 months, and 3 months. Notably, these field sera had significant higher OD_405_ value of IgG (against peptides or VLP of PCV2) at aged 1 day compared with that aged 1 month (*p* < 0.05). In the PCV2-negative pig (delivered by Caesarian-section and raised on sterile milk replacer) sera, no significant differences were observed between that OD_405_ value of IgG (against peptides or VLP of PCV2) at aged 1 week and 1 month (Fig. 1b). This implied that field piglets absorb large amount of antibodies after birth.

### Peptide-specific antibody response in mice

**Fig. 2:**
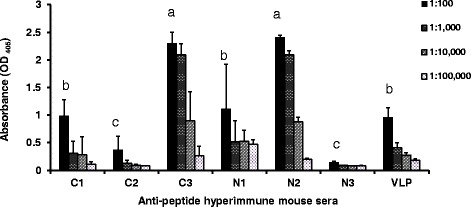
To evaluate the immunogenicity of each peptides, these peptides (C1, C2, C3, N1, N2, and N3) were conjugated with KLH as the immunogen to raise antiserum, respectively. Four mice per group were immunized subcutaneously with a given conjugated peptide or VLP of PCV2 three times at 2-week intervals. The anti‐peptide specific antibody titer was measured in each mouse serum by the iELISA. All samples were assayed twice. Each bar represents the mean ± SD of four mice sera. Treatments with different letters have statistically significant differences on that anti‐peptide specific antibody titer. Statistical significance was calculated using paired Student’s *t*-test. Significant *p* values are indicated as *p* < 0.05. Data shown were representative of one to two experiments

After three immunizations, the anti‐peptide specific antibody titer was measured in each mouse serum by the iELISA. Surprisingly, just C1, C3, N1, N2, and VLP of PCV2 were capable of inducing specific antibodies, but C2 and N3 were not (Fig. 2). Particularly, C3 and N2 elicited higher specific antibodies titer than other peptides or VLP of PCV2 did (*p* < 0.05). C1, N1, and VLP of PCV2 elicited higher specific antibodies titer than C2 or N3 did (*p* < 0.05). This results showed C3 or N2 that conjugated to KLH should be a good immunogen in murine immune responses. To make sure those antisera could recognize authentic viral protein, we performed IFA test to examine those antisera.

### Reactivity of PCV2 virus with various antisera

**Fig. 3:**
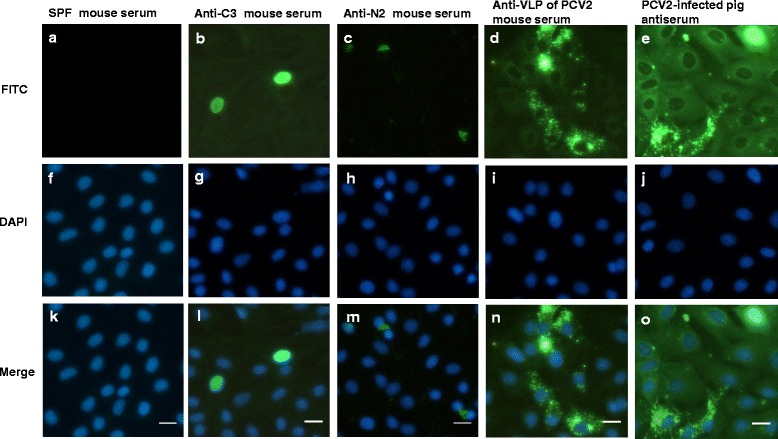
Localization of viral proteins of PCV2 by indirect IFA. Localization of viral proteins of PCV2 was assessed by indirect IFA using anti-PCV2 polyclonal antisera on the Porcine Circovirus Type 2 FA substrate slide (VMRD). Each column represents a different serum staining (**a, f,** and **k**) SPF mouse serum, (**b, g,** and **l**) anti-C3 mouse serum, (**c, h,** and **m**) anti-N2 mouse serum, (**d, i,** and **n**) anti-VLP of PCV2 mouse serum, (**e, j,** and **o**) PCV2 convalescent-phase swine antiserum. Upper row: Fluorescence microscopy of viral proteins of PCV2 were identified (*green*). Middle row: Nuclei were stained with DAPI (*blue*). Bottom column: The merge of the images. Scale bars, 20 μm. **b, c,** and **d** One representative image from a single experiment with a total of four mouse sera was shown. **e** One representative image from of a single experiment with a total of four pig sera was shown

These commercial FA substrate slides were PCV2 virus (strain: T657)-infected porcine kidney cells (PK) fixed on the surface of Teflon-masked slides. The positive signal was shown, as the epitope of PCV2 antigen exposure on infected cells were bound by antibodies examined. As PCV2-infected PK cells were stained with anti-C3 mouse serum, positive signals were displayed in the nucleus only (Fig. 3b and l). One mouse serum from the N2-immunized group was produced unusual cytoplasmic staining in PCV2-infected PK cells (Fig. 3c and m). The anti-VLP of PCV2 mouse serum produced dispersing granular and cytoplasmic staining with rare intranuclear staining in PCV2-infected PK cells (Fig. 3d and n). The PCV2 convalescent-phase swine antiserum produced strong intranuclear and cytoplasmic staining in PCV2-infected PK cells (Fig. 3e and o). However, PCV2-infected PK cells stained with other anti-peptide mouse sera or SPF mouse sera all showed negative results. This test demonstrated anti-C3 antibodies, anti-VLP of PCV2 antibodies, and anti-N2 antibodies can recognize authentic viral protein of PCV2 virus (strain: T657). The results also indicated that anti-C1 antibodies, and anti-N1 antibodies cannot recognize authentic viral protein of PCV2 virus (strain: T657), and implying these antibody binding residues should be quite different with epitope residues in this virus (strain: T657). Therefore, we suggested amino acid sequence of C1 (belonged to PCV2b-1A/1B-CP) should be different with the sequence of CP of this virus (T657).

### Detection of the ORF2 protein by western blot

**Fig. 4:**
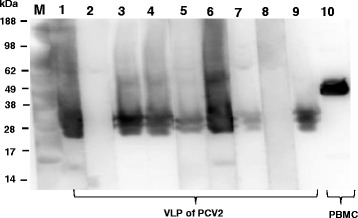
Imumunospecificities of mouse antisera were assayed against VLP of PCV2 (lanes 1–9) by Western blotting, using PBMC as a loading control (lanes 10). Lane 1, mouse was immunized with peptide of PCV2a CP; lane 2, mouse was immunized with peptide of ORF3 protein of PCV2 (negative control); lanes 3–6, mice were immunized with C3; lane 7 and 9, different mouse mAb against PCV2 (positive control); lane 8, SPF mouse serum was also used as negative control; lane 10, mouse mAb against β actin (control). Data shown are representative of two independent experiments with similar results

The reactivity of mouse anti-peptide serum was determined in a Western blot assay. The mAbs (positive control) gave a strong and specific reaction with proteins of approximately 27 or 30 kDa (Fig. 4). The band observed corresponded to the expected size of the manufacturer’s description of patent application (European Application EPO 05108299.8). We observed that anti-C3 mouse serum that gave strong reaction with proteins at 27 or 30 kDa (Fig. 4). The anti-C1 mouse serum showed no reactivity in the Western blot assay (data not shown). This results showed that anti-C3 antibodies can recognize the CP of PCV2 virus.

### Detection of swine anti-C3 specific IgA, IgG, and IgM

**Fig. 5:**
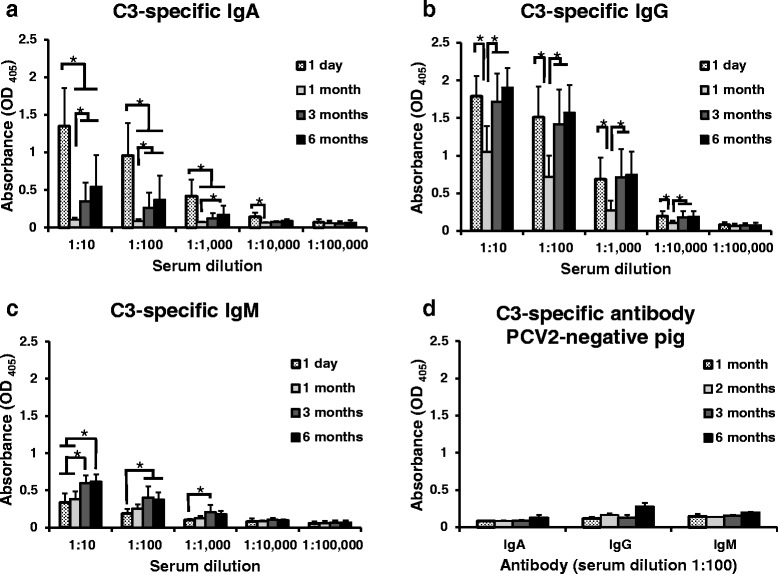
Detection of swine anti-C3 IgA, IgG and IgM in sera by iELISA. In the unvaccinated naturally PCV2-infected pig farm, 22 newborn piglets of TBP, were delivered from 11 sows during 4 seasons of 1 year. Blood samples were collected after parturition (after colostrum uptake), on the first day, the first month, the third month, and the 6th month of life, respectively. Serum samples from these pigs were used to detect anti-C3 specific antibodies by an iELISA. **a** Pig anti-C3 specific IgA were detected in these sera. **b** Pig anti-C3 specific IgG were detected in these sera. **c** Pig anti-C3 specific IgM were detected in these sera. **d** 16 PCV2-negative sera (4 each from 1-, 2-, 3-, and 6-month old pigs) were collected from the PCV2-negative herd in the primary specific pathogen free (SPF) pig facility. Serum samples from these pigs were used to detect anti-C3 specific antibodies (IgA, IgG, and IgM) by an iELISA. All samples were assayed twice. Each bar represents the mean ± SD of pig sera. Data shown were representative of two experiments. Significant p values are indicated as *p < 0.05. Statistical significance was calculated using paired Student’s *t*-test

Field sera collected from the PCV2-infected herds were used to detect anti-C3 specific antibodies by an iELISA. The results indicate that these pigs had the highest C3-specific IgA level on Day 1 during the first 6 months of life (*P* < 0.01) (Fig. 5a). These pigs had higher OD_405_ value of C3-specific IgG on Day 1, Month 3 and Month 6 than in Month 1 (Fig. 5b); furthermore, these pigs had higher C3-specific IgM level in Month 3 and Month 6 than on Day 1 (Fig. 5c). These positive sera had higher OD_405_ value of C3-specific IgG than C3-specific IgM (Fig. 5). One month postpartum, i.e. near weaning, OD_405_ value of C3-specific IgA levels and IgG levels have dropped 12-fold and 1.8-fold respectively (at 1:10 dilution) whereas IgM levels raised only slightly. Coincidentally, OD_405_ value of C3-specific IgA and IgG levels started to increase after aged 1 months (after weaning). This means piglets might be susceptible to PCV2 infection in this weaning period with low serum IgA and IgG levels.

In the PCV2-negative pig (delivered by Caesarian-section and raised on sterile milk replacer) sera, no significant differences were observed between that OD_405_ value of C3-specific IgA and IgM at aged 1 month, 2 months, 3 months, and 6 months (Fig. 5d). These PCV2-negative pig sera showed higher OD_405_ value of C3-specific IgG at aged 6 months compared with aged 1 month, 2 months, and 3 months. Notably, all PCV2-negative pig sera showed low OD_405_ value (<0.5) of C3-specific IgG, IgA, and IgM. It implied these PCV2-negative pig were not infected with PCV2.

### Comparison between amino acid residues of six PCV2 CP

**Fig. 6:**
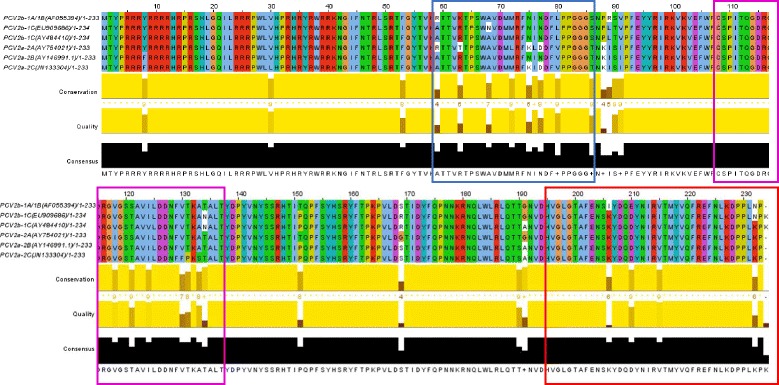
Sequence alignments of the CP of PCV2 strains from different PCV2 genetic clusters were used [22]. The amino acid residues of C1, C2, and C3 belong to PCV2b-1A/1B and locate in the blue box, magenta box, and red box, respectively. Sequence alignments were made using the T-Coffee multiple-alignment tool [32] and displayed with Jalview Version 2 [33]

As evident in Fig. 6 the variation in sequence is concentrated on residues 8, 30, 53, 59, 63, 68, 75, 76, 77, 88, 89, 134, 151 169, 191, 206, 210, 215, 232, and 233. Among PCV2a-CP and C1 (PCV2b-1A/1B-CP) were not completely homologous at peptide sequences, while there was only 79% (22/28) amino acid identity (Fig. 6). This means the amino acid residues (59–86) of C1 located at the variety region of CP of these diversity of PCV2 strains. Therefore, this might be one of antigenic differences among PCV2 strains. Among PCV2a-CP and C2 (PCV2b-1A/1B-CP) were most completely homologous at peptide sequences, while there was 97% (29/30) amino acid identity. Among PCV2a-CP and C3 (PCV2b-1A/1B-CP) were most completely homologous at peptide sequences, while there was 92% (36/39) amino acid identity.

### Location of amino acid residues of C1, C2, and C3 on the 3D model of the PCV2 CP

**Fig. 7:**
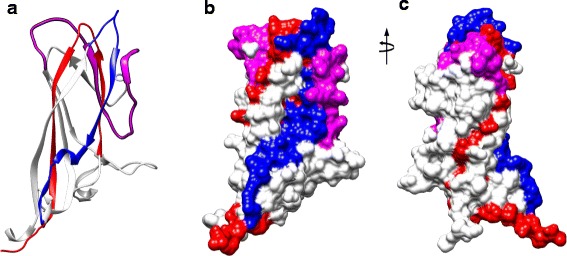
Location of amino acid residues of each designed peptide on the PCV2 CP. **a** The secondary structures of the single PCV2 CP (PDB accession no. 3R0R) is represented as ribbon diagram. **b** and **c** Three-dimensional model of the PCV2 capsid protein rendered by the Chimera software as a solid surface looking at two different view. The residues of C1, C2, and C3 are labeled in blue, magenta, and red, respectively

Based on the crystal structure of PCV2 CP (PDB codes 3R0R) was reported and using Chimera software to visualize spatially the location of amino acid residues of C1, C2, and C3. The secondary structures of the single PCV2 CP was represented as ribbon diagram (Fig. 7a). Three-dimensional model of the PCV2 CP rendered by the Chimera software as a solid surface looking at two different view (Fig. 7b and c). Notably, the residues of C2 are divided into two distinct domains. Most residues of C3 and C1 were present on the surface of PCV2 CP, however, some residues of C2 are not exposed surface of PCV2 CP, and non-C2 residues are on one of C2 domains. In the view of 3-D structure of the CP, the conformational epitope of CP that involved tertiary and quaternary structure which amino acid residues either inside or outside the CP. That might explain that the linear form of peptide (C2) was hardly to mimicking viral proteins of PCV2 in nature.

## Discussion

According to previous studies [22–26], we designed three peptides of PCV2 CP that located N-terminus (C1), middle region (C2), and C-terminus (C3) respectively. We pretested immunoreactivities of these peptides with sera from PCV2-infected pigs. The ELISA results showed these field sera had immunoreactivity with these peptides and CP. These data also showed the immunoreactivity of the field sera and C3 or N1 was stronger than that of the swine sera and other peptides. To demonstrate these peptides can mimic the epitopes present on the native PCV2, we utilized the conjugated peptide-KLH to inoculate mice and generate polyclonal antibody. Our data showed that C3 and N2 elicited higher specific antibodies titer than other peptides or CP did. Some peptides (C2 and N3) showed good immunoreactivities with sera from PCV2-infected pigs, however, they did not elicit good specific antibodies titer in mice. In addition, PCV2-infected PK cells were stained with mouse hyperimmune sera, positive signals were displayed different pattern distribution in the cell. As PCV2-infected PK cells were stained with anti-C3 mouse serum, positive signals were displayed a local distribution in the nucleus only. The anti-N2 mouse serum produced unusual cytoplasmic staining in PCV2-infected PK cells. The anti-VLP of PCV2 mouse serum produced dispersing granular and cytoplasmic staining and less intranuclear staining in PCV2-infected PK cells. The PCV2 convalescent-phase swine antiserum produced strong intranuclear and cytoplasmic staining in PCV2-infected PK cells. Since these different antisera were tested on the same FA substrate slide, the cellular localization of PCV2 viral protein and cellular alterations should be similar at the same time point post-infection. According to previous study, PCV2 entered the nucleus for replication and virus assembly and encapsidation occurred with the participation of the nuclear membrane [35]. They also indicated that immature virions left the nucleus and formed intracytoplasmic inclusion bodies in a second cytoplasmic phase. These phenomenon were also demonstrated by using anti-inactivated PCV2a virus (Circovac®, Merial) mouse sera, anti-inactivated chimeric PCV1/2 (Fostera™, Pfizer) mouse sera, anti-VLP of PCV2 (CircoFLEX®, Boehringer Ingelheim) mouse sera, and anti-VLP of PCV2 (Porcilis®, Intervet) mouse sera on the same FA substrate slide (Additional file 1). Therefore, we suggested that the tertiary or linear form C-terminal sequence (C3) of PCV2 capsid peptide mainly appeared in the nucleus locally, VLPs of PCV2 appeared in the cytoplasm mainly, and other ORF proteins of PCV2 might be shown in cytoplasm or nucleus. That could be different results on different cell line or at different time point’s post-infection [35].

Among this PCV2 virus (strain: T657) and C1 were supposed to have not completely homologous at peptide sequences of CP. We observed that anti-PCV2a CP peptides (P64) mAbs that produced strong intranuclear positive staining in PK cells infected with this PCV2 strain (unpublished observations). Among C1 (PCV2b-1A/1B-CP) and PCV2a-CP were not completely homologous at peptide sequences, while there was only 79% (22/28) amino acid identity (Fig. 6). This means the amino acid residues (59–86) of C1 located at the variety region of capsid protein of these diversity of PCV2 strains. Therefore, this might be one of antigenic differences among PCV2 strains. In contrast to C1, among C3 (PCV2b-1A/1B-CP) and PCV2a-CP were most completely homologous at peptide sequences, while there was 92% (36/39) amino acid identity. For this reason, just mouse anti-C3 mouse serum reacted with this PCV2 strain (T657). Since propagation of PCV2 in cell culture is often difficult [36–38], we used the homemade IFA slide composed of PCV2b-infected PBMC cells collected from conventional piglets. IFA reactivity of anti-C1, anti-C3, and anti-N1 antisera produced positive signals in PCV2b-infected PBMC (data not shown). The anti-C1 antiserum also showed specific reaction with recombinant capsid protein of PCV2b in the Western blot assay (data not shown).

Published data have demonstrated that some epitopes in C2 (residues 108–136), especially residues 117–131(B-133) determined by PEPSCAN analysis was more effective for detection of anti-PCV2 antibodies [23]. Therefore, B-133 was considered as a serological marker for experimental and natural infection [39]. The crucial residues 130, 133 of PCV2 CP are responsible for the differential reactivity of mAbs to different PCV2 strains [22]. The core region (residues 119–128) could be deduced as a core component of the epitope by use of a random peptide-displayed library and polyclonal antibody [40]. The peptide P122–136 (AVILNDNFVTKATAL) was confirmed to contain the dominant B-cell epitope by dot-ELISA and peptide ELISA [41]. Based on these published data, we suggested that the peptide C2 could be a promising immunogen candidate in mice. However, they didn’t elicit any specific antibodies in this study. It is probably that the linear form of C2 could not be folded into two distinct domains as the native structure of PCV2 CP (Fig. 7). Notably, some residues were not exposed on the surface of the viral particle, and non-C2 residues were on one of C2 domains (Fig. 7b). These contributed the complexity of the native structure of PCV2 CP and immunogenicity.

In contrast, most residues of C1 and C3 were present on the surface of PCV2 CP, in the view of 3-D structure of the capsid protein. These peptides might mimic the epitopes present on the native PCV2 CP to elicit specific antibodies in mice successfully. Some data are worth noting about the non-PCV2 CP, such as N3 did not elicit any specific antibodies in mice, even it was constituted by the full length of PCV2 ORF 9 protein. Although N3 could be reacted with field sera collected from the PCV2-infected herds, but it hardly elicited any antibody responses in mice. It might be due to the immunogenicity of peptides or different adaptive immune response in different host animal.

Our data showed that PCV2-infected herd had higher OD_405_ value of C3-specific IgG at aged one day, 3 months, and 6 months, compared with aged one month (Fig. 5b). These pigs had higher C3-specific IgA level at aged 1 day than aged 1 month (Fig. 5a). The same sera samples from these pigs were also used to measure the total globulin of pig sera by automated analysis equipment. This phenomenon is very similar to the result of distribution of the total globulin of pig sera (Additional file 2). According to previous study, almost all conventional sows were seropositive for PCV2 [42] and the majority of newborn piglets received colostral antibodies from seropositive sows and had various levels of maternally derived antibodies. Our finding is in agreement with this point. Our data showed that this field sera had higher OD_405_ value of IgG (against peptides or VLP of PCV2) at either 1 day, or one month of age than that of PCV2-negative pig (piglets were delivered by Caesarian-section and send into sterile isolation cage and raised on sterile milk replacer) sera at aged 1 week or 1 month (*P* < 0.05) (Fig. 1). This suggested that suckling newborn piglets absorb maternal transferring antibodies (C3-specific IgG and IgA) from colostrum and milk in the first 24 h, since sow do not transfer maternal antibodies to their fetuses in utero [43]. The changes in serum globulin may reflect the extreme changes at 1 month after birth since it’s time near weaning and continue to decay most of globulins which were absorbed from colostrum or milk. Piglets were showed to develop the adaptive immune response as evidenced by increase synthesis of globulin at aged 3 months or after weaning.

This study used a reverse vaccinology approach [30] to demonstrate the ideal immunogens relevant for PCV2 peptide vaccine development, initiating from identification of peptides reacting well with sera from PCV2-infected herds. Subsequently, those selected peptides were used as immunogens to immunize the mice. This study showed that mouse anti-PCV2 antisera could be generated by specific synthetic peptides and recognizing PCV2 viral protein. Also, peptide C3 indeed mimic PCV2 CP is capable of inducing antibody response. Previous reports indicated that the residues 230–233 [24] or the residues 231–233 [25] participate in the formation of conformational epitope and is suggested as the part of neutralizing epitopes of PCV2. These residues were part of designed peptide (C3). Our study demonstrated the C3 not only owned the antigenicity, but also possessed immunogenicity. It is the serological marker for the immune response of PCV2 infection.

## Conclusions

Our study demonstrated the specific peptides (C1, C3, N1, and N2) contribute to immunogenic potential of mimicking viral proteins of PCV2. We figured out some specific synthetic peptides (C3 and N2) could mimic viral proteins of PCV2 in nature. This study also focused on the nature of the binding between the antigen (peptides or viral protein) and antibodies which come from either pig sera or anti-peptide mouse sera. Moreover, our results indicated that suckling newborn piglets absorb high concentration of maternal transferring antibodies (C3-specific IgA and IgG) from colostrum and milk in the first 24 h, and it might protect themselves from PCV2 infection during neonatal age. Taken together, those discovers would be facile to generate antibodies by utilizing those peptides (C1, C3, N1, and N2). Further study will utilize these findings to reveal the interaction between epitope of viral proteins and antibodies, then to construct new peptide-base vaccine in future.

## Additional files

Additional file 1: Localization of viral proteins of PCV2 by indirect IFA. Localization of viral proteins of PCV2 was assessed by indirect IFA using anti-PCV2 polyclonal antisera on the Porcine Circovirus Type 2 FA substrate slide (VMRD). Each figure represents a different antiserum staining (a) anti-inactivated PCV2a virus (Circovac®, Merial) mouse serum, (b) anti-inactivated chimeric PCV1/2 (Fostera™, Pfizer) mouse serum, (c) anti-VLP of PCV2 (CircoFLEX®, Boehringer Ingelheim,) mouse serum, (d) anti-VLP of PCV2 (Porcilis®, Intervet) mouse serum, (e) anti-C3 mouse serum, and (f) PCV2 convalescent-phase swine antiserum. Nuclei were stained with DAPI (blue). Scale bars, 20 μm. a, b, and d One representative image from a single experiment with a total of two mice sera was shown. c and e One representative image from a single experiment with a total of four mice sera was shown. f One representative image from of a single experiment with a total of four pig sera was shown. (PDF 85 kb)

Additional file 2: The total globulin of pig sera were measured at different age. These sera were measured serum globulin concentration by automated analysis equipment (Hitachi 7170 analyzer, Japan). This study involved 22 newborn piglets of TBP, were delivered from 11 sows during 4 seasons of 1 year. Each dot represents the serum sample from different individual pig at different age. Blood samples from each pig were collected 4 times during this experiment: on the 1st day, 1st month, 3rd month, and 6th month of life, and the same colored dot represents the serum sample from the identical pig. Black horizontal bars represent median values, and significant *p* values are indicated as *p < 0.05. Statistical significance was calculated using paired Student’s *t*-test. (PDF 32 kb)

## Abbreviations

CP: Capsid protein
ECL: Enhanced chemiluminescence
iELISA: Indirect enzyme-linked immunosorbent assay
ORFs: Open reading frames
PBST: PBS containing 0.05% Tween 20
PCV: Porcine circovirus
PCV1: Porcine circovirus type 1
PCV2: Porcine circovirus type 2
PDNS: Porcine dermatitis and nephropathy syndrome
PMWS: Post-weaning multisystemic wasting syndrome
SPF: Specific pathogen free
VLP: Virus-like particle.
