# Supplementary figures and images for: Peptides mimicking viral proteins of porcine circovirus type 2 were profiled by the spectrum of mouse anti-PCV2 antibodies
Source: BMC Immunol. 2017 May 15;18:25. doi: 10.1186/s12865-017-0211-2 (PMC5433044; doi:10.1186/s12865-017-0211-2)

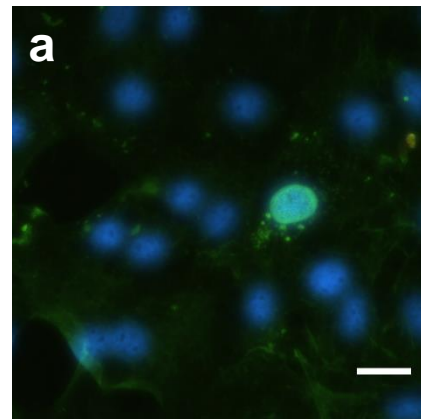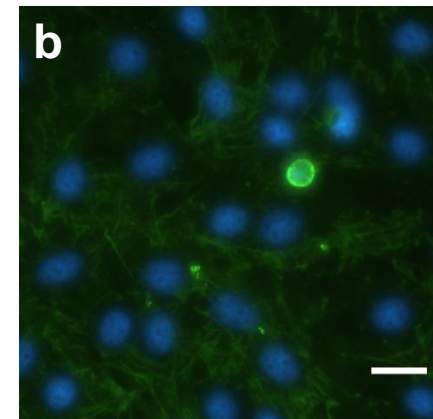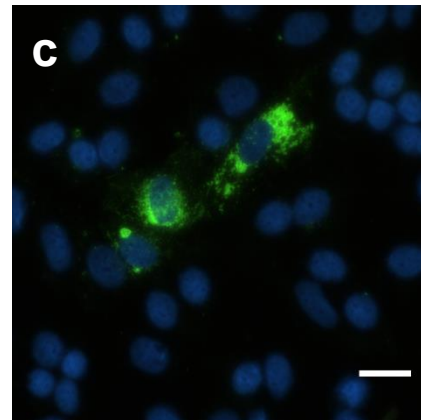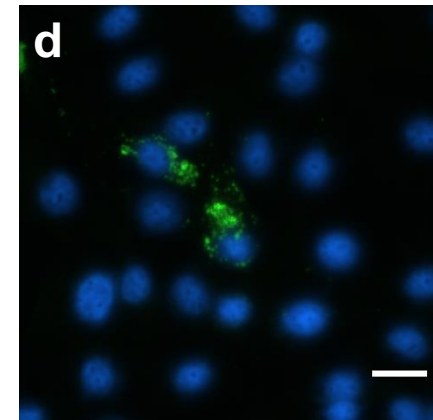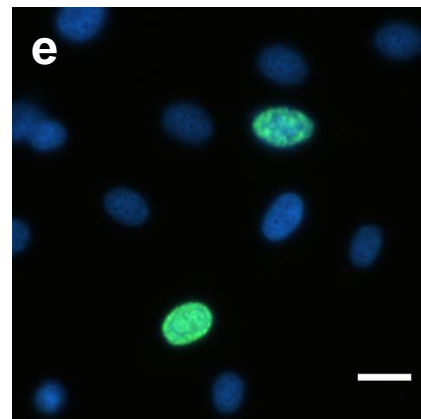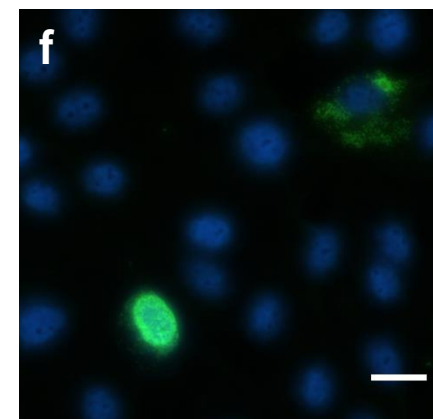

Supplement: Supplementary file 1 — Localization of viral proteins of PCV2 by indirect IFA. Localization of viral proteins of PCV2 was assessed by indirect IFA using anti-PCV2 polyclonal antisera on the Porcine Circovirus Type 2 FA substrate slide (VMRD). Each figure represents a different antiserum staining (a) anti-inactivated PCV2a virus (Circovac®, Merial) mouse serum, (b) anti-inactivated chimeric PCV1/2 (Fostera™, Pfizer) mouse serum, (c) anti-VLP of PCV2 (CircoFLEX®, Boehringer Ingelheim,) mouse serum, (d) anti-VLP of PCV2 (Porcilis®, Intervet) mouse serum, (e) anti-C3 mouse serum, and (f) PCV2 convalescent-phase swine antiserum. Nuclei were stained with DAPI (blue). Scale bars, 20 μm. a, b, and d One representative image from a single experiment with a total of two mice sera was shown. c and e One representative image from a single experiment with a total of four mice sera was shown. f One representative image from of a single experiment with a total of four pig sera was shown. (PDF 85 kb) [file 12865_2017_211_MOESM1_ESM.pdf]

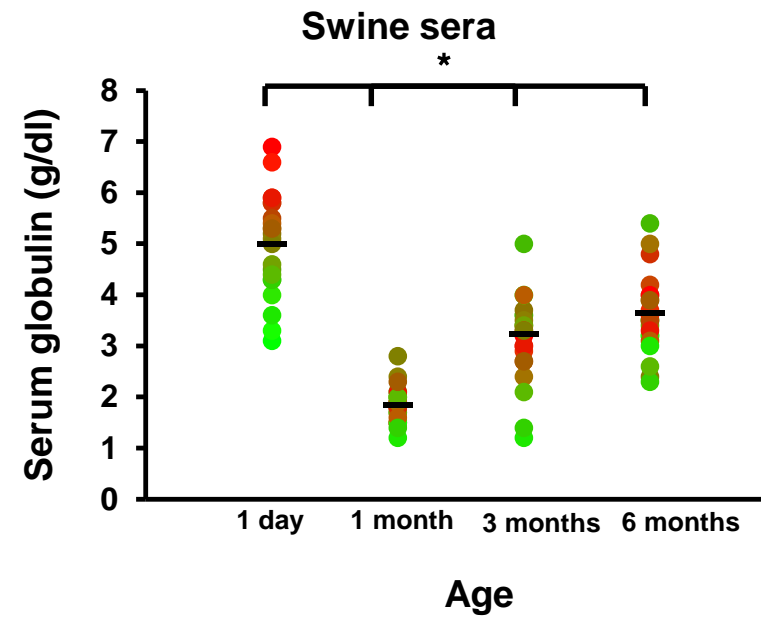

Additional File 2 (Hung et al.)

Supplement: Supplementary file 2 — The total globulin of pig sera were measured at different age. These sera were measured serum globulin concentration by automated analysis equipment (Hitachi 7170 analyzer, Japan). This study involved 22 newborn piglets of TBP, were delivered from 11 sows during 4 seasons of 1 year. Each dot represents the serum sample from different individual pig at different age. Blood samples from each pig were collected 4 times during this experiment: on the 1st day, 1st month, 3rd month, and 6th month of life, and the same colored dot represents the serum sample from the identical pig. Black horizontal bars represent median values, and significant p values are indicated as *p < 0.05. Statistical significance was calculated using paired Student’s t-test. (PDF 32 kb) [file 12865_2017_211_MOESM2_ESM.pdf]
